# Preharvest UVB Application Increases Glucosinolate Contents and Enhances Postharvest Quality of Broccoli Microgreens

**DOI:** 10.3390/molecules26113247

**Published:** 2021-05-28

**Authors:** Yingjian Lu, Wen Dong, Tianbao Yang, Yaguang Luo, Pei Chen

**Affiliations:** 1 College of Food Science and Engineering, Nanjing University of Finance and Economics, Nanjing 210095, China; yingjianlu@nufe.edu.cn; 2 Beltsville Agricultural Research Center, Food Quality Laboratory, U.S. Department of Agriculture, Agricultural Research Service, Beltsville, MD 20705, USA; hanywenwen1986@gmail.com (W.D.); Yaguang.Luo@ARS.USDA.GOV (Y.L.); 3 Beltsville Agricultural Research Center, Environmental Microbial & Food Safety Laboratory, U.S. Department of Agriculture, Agricultural Research Service, Beltsville, MD 20705, USA; 4 Beltsville Human Nutrition Research Center, Methods and Application of Food Composition Laboratory, U.S. Department of Agriculture, Agricultural Research Service, Beltsville, MD 20705, USA; pei.chen@usda.gov

**Keywords:** *Brassica oleracea* var. *italica*, phenolics, functional food, calcium, UVB radiation

## Abstract

Broccoli microgreens have shown potential health benefits due to their high glucosinolate (GL) levels. Previously, we observed that postharvest UVB treatment did not have much effect on increasing GLs in broccoli microgreens. In this study, we investigated the influence of preharvest UVB irradiation on GL levels in broccoli microgreens. UHPLC-ESI/ITMS analysis showed that preharvest UVB treatments with UVB 0.09 and 0.27 Wh/m^2^ significantly increased the glucoraphanin (GLR), glucoerucin (GLE), and total aliphatic GL levels by 13.7 and 16.9%, respectively, in broccoli microgreens when measured on harvest day. The nutritional qualities of UVB-treated microgreens were stable during 21-day storage, with only small changes in their GL levels. Broccoli microgreens treated before harvest with UVB 0.27 Wh/m^2^ and 10 mM CaCl_2_ spray maintained their overall quality, and had the lowest tissue electrolyte leakage and off-odor values during the storage. Furthermore, preharvest UVB 0.27 Wh/m^2^ treatment significantly increased GL biosynthesis genes when evaluated before harvest, and reduced the expression level of myrosinase, a gene responsible for GL breakdown during postharvest storage. Overall, preharvest UVB treatment, together with calcium chloride spray, can increase and maintain health-beneficial compound levels such as GLs and prolong the postharvest quality of broccoli microgreens.

## 1. Introduction

In recent years, consumption of microgreens has increased as a result of consumers’ appreciation for their diverse flavors, beautiful colors, and highly nutritional value. Microgreens are seedlings of edible plants harvested around two weeks after germination at the emergence of the first true leaves [1]. Previous studies have shown that broccoli microgreens contain higher glucosinolate (GL) content than broccoli florets and mature leaves, indicating that microgreens are a richer source of GLs than mature broccoli [2]. Glucoraphanin (GLR) is the most abundant GL in broccoli heads, making up over 50% of the total GLs in broccoli heads, followed by glucoerucin (GLE) and glucoiberin [3,4]. GL hydrolysis products, especially sulforaphane (SFR) and erucin (ERC), have received much attention due to their chemopreventive capabilities [5]. Many studies have revealed that glucosinolates/isothiocyanates can potentially reduce the risk of chronic disease [6,7,8].

Environmental stresses can significantly influence the content of biologically active molecules in plants. Most notably, abiotic stresses, such as salinity, UVB, temperature, air pollution, heavy metal, and mechanical wounding, lead to changes in the secondary metabolism of plants [9]. For instance, flavonoids that are common in the plant kingdom are produced in response to almost all abiotic stresses [10]. In addition, UVB activates wound- and defense-signaling pathways that can cause an accumulation of secondary plant metabolites, such as flavonoids and other phenolic compounds [11]. GL levels in Brassica vegetables are determined mostly by genetic factors; however, abiotic stresses such as salinity and temperature can also influence the GL levels [2,12,13]. Postharvest exposure to a low UVB dose of up to 0.54 KJ/m^2^ induced a distinct increase in major GL derivatives in broccoli sprouts [2,14]. Heinze et al. [15] also reported that the exposure of Pal Choi (*Brassica rapa* subsp. *chinensis*) to reduced UVB dosages elevated the levels of GLs. Thus, the increase in GL levels in the secondary plant metabolism by UVB treatments have beneficial implications for humans. However, the effect of preharvest UVB radiation on shelf life and the levels of two major GLs, namely GLR and GLE, in broccoli microgreens has never been studied.

In Brassica vegetables, all of the GLs are synthesized from amino acids, for example, aliphatic glucosinolates from methionine, aromatic glucosinolates from phenylalanine, and indolic glucosinolates from tryptophan [16,17,18,19,20,21]. Two genes, methylthioalkylmalate synthase (*MAM*) and cytochrome (*CYP79/CYP83*), play important roles in the regulation of different stages of GLs synthesis. MAM catalyzes amino acid elongation, which serves as the substrate for GL synthesis. The CYP gene family plays an important role in synthesizing the core GLs from chain elongated methionine [21]. GLs are hydrolyzed by myrosinases (MY) to release glucose groups, and form isothiocyanates, thiocyanates, nitriles, etc. This breakdown is involved in defense signaling. Calcium treatment has been shown to increase both aliphatic and indolic GL levels, as well as the yield and shelf life of broccoli microgreens under both natural and stress conditions [1,22,23]. However, GL gene regulation in response to UVB treatment has not previously been studied.

In this study, we evaluated the effects of preharvest UVB treatment with and without CaCl_2_ spray on GL content and shelf life of broccoli microgreens, and the expression patterns of three key genes (*MAM*, *CYP79F1*, *MY*) involved in GL biosynthesis and catabolism. 

## 2. Materials and Methods

### 2.1. Seed Germination and Treatments

The seeds of broccoli (*Brassica oleracea* var. *italica*) cultivar ‘De Cicco’ were obtained from a local seed store (Silver Spring, MD, USA) and grown in a plant growth chamber. The seed germination and further treatments followed previous protocol, as now described [1]. Seeds were sowed evenly on a hydroponic pad soaked with acidified water (pH 5.6) and set in the dark for 4 days. On the fifth day, seedlings were exposed to light at an intensity of 42 µmol·s^−1^·m^−2^ light intensity for a 12 h photo period until harvest on day 10 after sowing. Broccoli seedlings were subjected to two different UVB levels: 0.09 and 0.27 W h/m^2^ using a UVB fluorescent light source (312 nm) for an additional 2 h per day. The seedlings were sprayed once a day with water only (control) or 10 mM CaCl_2_. Microgreens were harvested by cutting the hypocotyl about 1 cm above the pad surface, and 10 g portions were sealed in 5 cm × 5 cm sealed bags. Samples for postharvest evaluation were stored at 5 °C. All others were frozen at −80 °C.

### 2.2. Extraction of Glucosinolates 

A modified procedure was used for GL extraction from microgreen samples [2]. First, frozen microgreen samples (200 mg) were lyophilized for 48 h and milled into fine powders. Next, the powders were dissolved in 5 mL of 60% methanol–water and sonicated for 30 min at 70 °C. The extracts were then cooled and centrifuged at 10,000× *g* for 15 min at 4 °C, and the final supernatant was filtered through a Waters 0.45 μm nylon filter (Milford, MA, USA) for further ultra-high-performance liquid chromatography with electrospray ionization (UHPLC-ESI)/ion-trap mass spectrometry (ITMS) analysis.

### 2.3. UHPLC-ESI/ITMS

An LC/MS system consisting of an Agilent 1200 UHPLC system with a diode array detector (DAD) (Agilent Technologies, Palo Alto, CA, USA) was used in combination with an LCQ Deca ion-trap mass spectrometer (Thermo Fisher Scientific Inc., Waltham, MA, USA). The temperature of the reverse phase C18 column (2.1 mm × 150 mm, 3.5 μm) (Waters, Milford, MA, USA) was set to 40 °C.

We used a Milli-Q system (Millipore Lab., Bedford, MA, USA) to obtain HPLC-grade water. HPLC-grade methanol, formic acid, acetonitrile, and ethyl acetate were purchased from VWR International, LLC (Clarksburg, MD, USA). The glucoraphanin potassium salt and glucoerucin potassium salt were bought from Sigma-Aldrich Corporation (St. Louis. MO, USA). The UHPLC gradient method was set as follows: Mobile phase A: 0.1% formic acid in H_2_O; Mobile phase B: 0.1% formic acid in acetonitrile. The starting percentage of mobile phase B was 2%; this then changed linearly to 30% in 20 min, elevated to 90% in 25 min, and back to 2% in 26 min. The gradient was then washed at 95% B for 5 min and returned to its initial condition (2% B) for 5 min to re-equilibrate the column for the next injection. The injection volume was 20 µL and the flow rate was 0.7 mL/min. The detecting wavelength was set to 229 nm [22].

The full scan mode of MS (100 and 700 *m*/*z*) was utilized to identify MS peak. ESI in negative ion was determined, and the ESI/ITMS parameters were followed below: sheath gas flow rate, 80; aux and sweep gases, 15; spray voltage, 3.5 kV; heated capillary temperature, 250 °C; capillary voltage, 4.0 V; tube lens offset, 20 V. MS spectra were collected from 0 to 26 min.

### 2.4. Qualitative and Quantitative Analysis of GLs

Glucoerucin and glucoraphanin were determined by their UV-visible chromatography, MS spectra, and the order of elution time from similar acquisition conditions as described [2]. The levels of glucoerucin and glucoraphanin were quantified by comparing with the integrating peak areas of a range of known concentrations of commercial standards. The Xcalibur 2.4 software (Thermo Fisher Scientific Inc., Waltham, MA, USA) was used to calculate the integrating peak areas.

### 2.5. Postharvest Quality and Physiological Assessment

The concentrations of carbon dioxide and oxygen in the headspace of sealed microgreen packages were measured using Checkmate II gas analyzer (PBI Dansensor Co., Denmark). Overall visual qualities were evaluated by ten highly trained people [24]. Off-odor was scored on a 1–5 scale, where 1 = no off-odor, and 5 = extremely strong off-odor. Overall quality was evaluated with a 9-point hedonic scale, where 9 = like extremely, 5 = neither like nor dislike and 1 = dislike extremely [25].

The tissue electrolyte leakage (TEL) was determined as described [1]. Briefly, threegram broccoli microgreens samples were immersed in distilled water for 30 min at 5 °C. The electric conductivity of each sample was measured by the Orion Model 135A conductivity meter (Orion Research Inc., Franklin, MA, USA). Total sample conductivity was determined on the same solutions after freezing at −20 °C for 24 h and subsequent thawing. Tissue electrolyte leakage was expressed as a percentage of the total sample conductivity.

### 2.6. Quantitative RT-PCR (qRT-PCR) Analysis

The total RNA was isolated from broccoli microgreen samples using the RNeasy Plant Mini kit (Qiagen, Germantown, MD, USA). The total RNA was quantified by a NanoDrop 1000 spectrophotometer (Thermo Scientific, Waltham, MA, USA). One μg total RNA samples were used for reverse transcription using the iScript™ RT kit (Bio-Rad, Hercules, CA, USA). The primer pairs, 5′-GGAGTTAGACGAAGTGGTGGGA-3′/5′-TGTGGCTACCTTTGGGAATGA-3′, 5′-AAGGTCGTCTGAAAGAGTTGGG-3′/5′-TGATTTCGTTGTCGTTAGTGCC-3′, 5′-GGTACATGGAGCCGCTAACA-3′/5′-TTTGGCTGGGCGTATTGAGT-3′, and 5′-CCAGAGGTCTTGTCCAGCCATC-3′/5′-GTTCCACCACTGAGCACAATGTTAC-3′ were used in the expression analysis of *CYP79F1* (GenBank No. KP693683), *MAM* (GenBank No. AF399834.2), *MY* (GenBank No. EU004075) and *BoACT* (GenBank No. AF044573), respectively. RT-qPCR was performed with the following thermal cycle setting: 95 °C for 2 min, followed by 40 cycles of 95 °C for 5 s and 60 °C for 15 s. The relative expression levels were calculated based on the 2^−∆∆CT^ method [26], normalized by a housekeeping gene *ACTIN*, *BoACT*, and expressed as fold changes (the lowest value = 1).

### 2.7. Statistical Analysis

All the datasets are expressed as average ± standard deviation (*n* = 3). Differences between means were determined by analysis of variance (ANOVA) with Tukey’s HSD post hoc test (*p* < 0.05).

## 3. Results and Discussion

### 3.1. Glucosinolate Levels of Microgreens during Cold Storage

Our previous study showed that the preharvest 10mM calcium spray could significantly increase plant GL levels [2]. Thus, we applied one factor (calcium chloride: 10 mM) by two factor (UVB: 0.09 and 0.27 W h/m^2^) treatments on the broccoli microgreens in this study. On harvest day (Day 0), broccoli microgreens treated with 10 mM CaCl_2_ and UVB 0.27 Wh/m^2^ showed the highest GLE, GLR, and total aliphatic GLS contents at 48.86, 2.01, and 50.87 µmol/g DW, respectively (Table 1). Compared to the negative control (without any treatment), treatments combining 10 mM CaCl_2_ and UVB elevated GLE and total aliphatic GLs by 61–69%, followed by calcium only (35% increase in GLs) and UVB only (~20% increase in GLs). These results indicate that preharvest calcium and UVB treatments stimulated GL accumulation in broccoli microgreens.

Furthermore, the control GLE and GLR levels decreased up to 56% from Day 0 to 21 during storage. However, all UVB-treated microgreens showed slower reduction rates (less than 30%) of GLs than other treatments. In the first week of storage, GLs after UV treatment decreased by less than 5% of the GL levels. Specifically, GLE in CaCl_2_ plus UV 0.09 Wh/m^2^ and CaCl_2_ plus UV 0.27 Wh/m^2^ -treated microgreens decreased from 46.60 and 48.86 µmol/g on Day 0 to 45.64 and 47.15 µmol/g on Day 3, and 45.01 and 45.46 µmol/g on Day 7, respectively. A similar trend was found for GLR in UVB-treated samples during the 7-day storage. On Day 21, 10 mM CaCl_2_ + UV0.09 and 10 mM CaCl_2_ + UV0.27-treated microgreens contained 34.44 and 36.15 µmol/g of GLE, respectively, on Day 21, which was only reduced by 26.1% and 26.0% from levels observed on Day 0. Overall, the nutritional quality of all UVB-treated microgreens was relatively stable during the entire storage period (21 days) and especially during the second-week storage, with only minor changes in terms of GL content. 

### 3.2. Effects of UVB Treatments on Microgreen Postharvest Quality Traits

**Figure 1:**
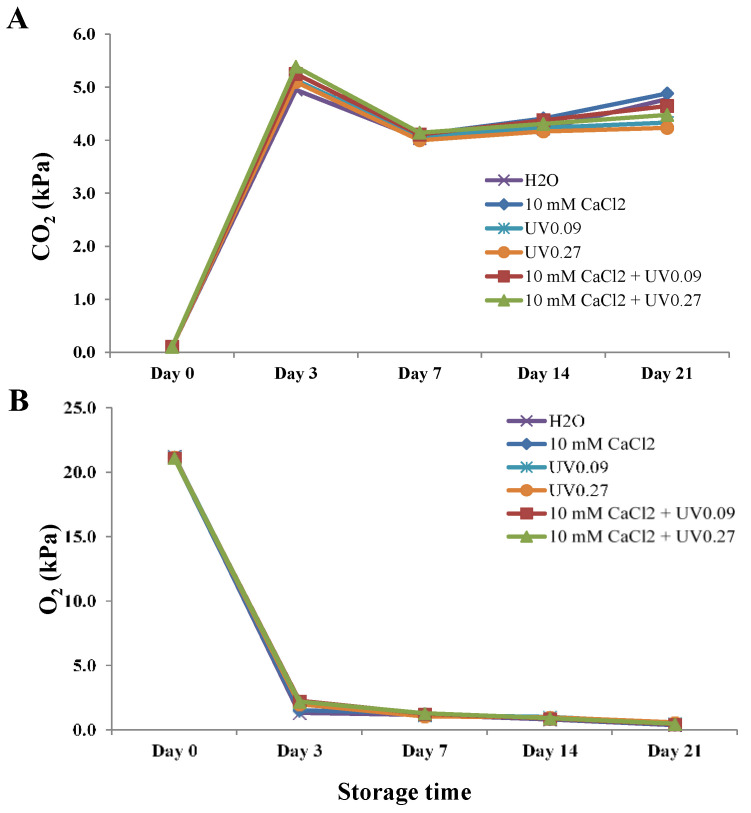
Effects of preharvest treatments on CO_2−_ (**A**) and O_2−_ (**B**) partial pressure within package on broccoli microgreens during cold storage (4 °C). UV0.09: 0.09 Wh/m^2^ UVB; UV0.27: 0.27Wh/m^2^ UVB. Data presented are the means of three replicates.

The CO_2_ partial pressures in all treatments dramatically increased from Day 0 through 3, decreased gradually from Day 3 to Day 7, and then maintained a steady level from Day 7 to the end of storage. There were no significant differences in CO_2_ partial pressures among different treatments and control (Figure 1A). 

The oxygen-partial pressures in water-control packages decreased rapidly from 21.0 kPa to 1.3 kPa on Day 3 and continued to decline steadily to 0.4 on Day 21 (Figure 1B). Samples treated with 10 mM CaCl_2_ plus UV showed significantly higher oxygen-partial pressures (2.0–2.2 kPa) than controls on Day 3. However, there were no statistical differences in oxygen-partial pressures among treatments at other time points during storage.

**Figure 2:**
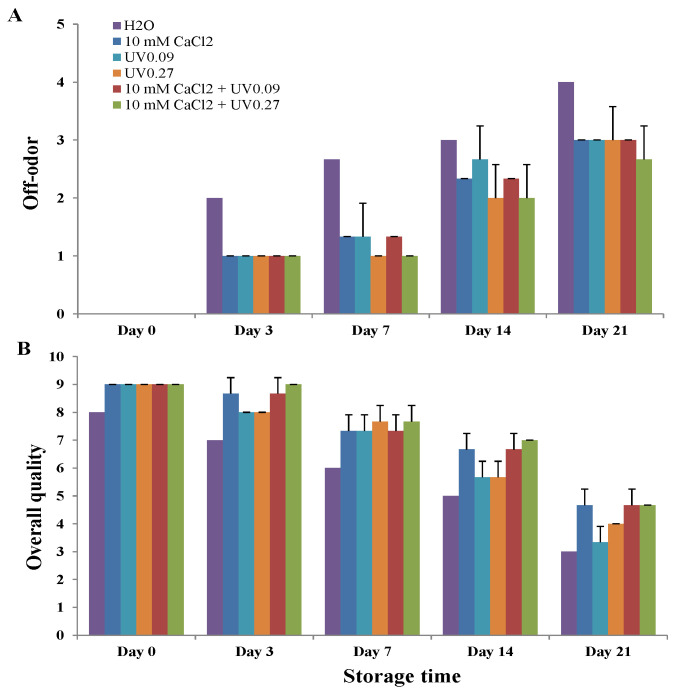
Effects of preharvest treatments on off-odor (**A**) and overall quality (**B**) of broccoli microgreens during cold storage (4 °C). UV0.09: 0.09 Wh/m^2^ UVB; UV0.27: 0.27Wh/m^2^ UVB. Data presented are the means of three replicates; vertical lines represent standard deviation.

We further examined the off-odor and overall visual quality for each treatment. Water-only treated seedlings yielded highest off-odor scores throughout storage (Figure 2A), and worst overall quality (Figure 2B). All calcium and UVB-treated samples had less off-odor than water-treated samples. Among them, the samples treated with 10 mM CaCl_2_ plus UVB 0.27 Wh/m^2^ had the least off-odor from Day 7 to Day 21, and best overall visual quality from Day 3 to Day 21. The results indicate that the microgreens treated with UVB 0.27 Wh/m^2^ after calcium spray retained the best postharvest quality and the longest shelf life, and were the only samples still acceptable (having an overall quality score of at least 7.0) on Day 14 of storage.

**Figure 3:**
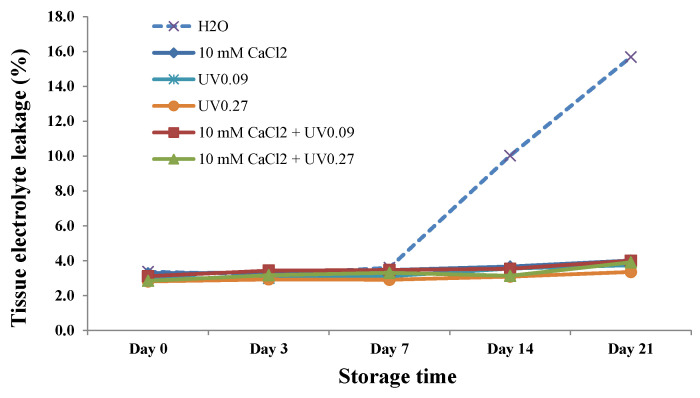
Effect of preharvest treatments on tissue electrolyte leakage of broccoli microgreens after 0, 3, 7, 14, and 21 d of storage at 4 °C. UV0.09: 0.09 Wh/m^2^ UVB; UV0.27: 0.27Wh/m^2^ UVB. Data presented are the means of three replicates.

We further measured TEL (total electrolyte leakage), which is closely related to tissue damage and fresh produce shelf life [23]. TEL in the water-only control increased sharply from Day 7 to Day 21, namely from 3.2 to 15.1% (Figure 3). In contrast, all preharvest UVB and calcium spray-treated microgreens significantly reduced the electrolyte leakage during the 21-day storage, and TEL increased only slightly from 3.0 to 3.6%. In general, the 10 mM CaCl_2_ with UVB 0.27 Wh/m^2^ treatment was the most effective for maintaining fresh appearance and overall quality throughout the storage period. Additionally, the lowest TEL values and off-odor scores were observed for these samples during the 21-day storage.

### 3.3. Expression Changes of Glucosinolate Biosynthetic and Breakdown Genes during Storage

**Figure 4:**
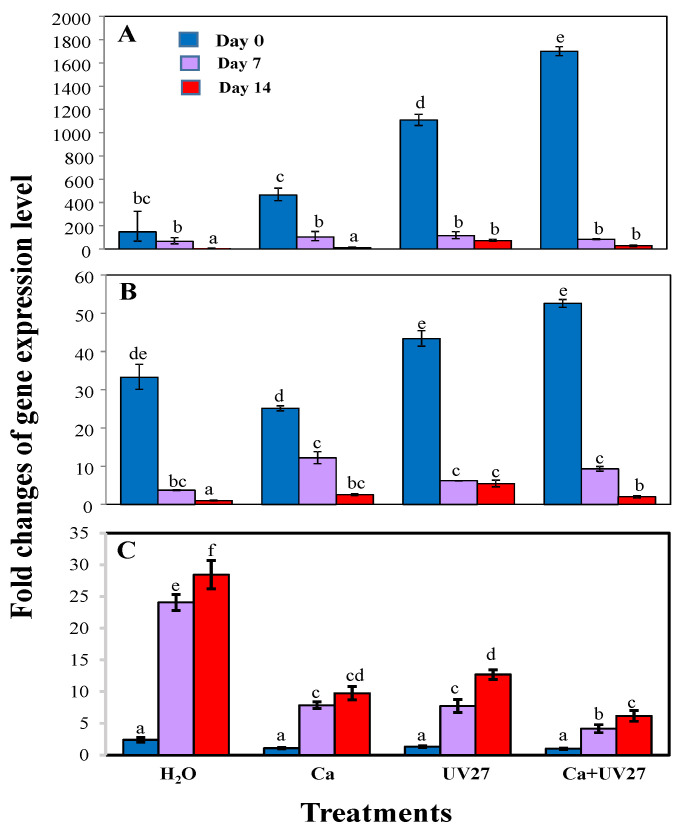
Expression patterns of glucosinolate structural genes *CYP79F1* (**A**), *MAM* (**B**) and *MY* (**C**) during postharvest storage. Ca: 10 mM CaCl_2_; UV27: 0.27Wh/m^2^ UVB. Total RNA was extracted from microgreens on Day 0 (harvest day), Day 7, and Day 14. *ACTIN* was used as the internal control. Error bars represent standard error of the mean. Different letters indicate significant differences among mean values (*p* < 0.05; *t*-test). Data presented are the means of three replicates.

We investigated the changes in expression of two key genes, *MAM* and *CYP79F1,* in the glucosinolate biosynthetic pathway from Day 0 to Day 21. On Day 0, the expression levels of *MAM* and *CYP79F1* in microgreens treated with UVB 0.27 Wh/m^2^ with 10 mM CaCl_2_ spray were significantly higher than others. For example, *CYP79F1* and *MAM* expression levels were higher by 12 and 10 fold, respectively, than for the water-only control (Figure 4A,B). During cold storage, the expression of *MAM* and *CYP79F1* was reduced significantly in all treatments. However, the expression levels of *MAM* and *CYP79F1* in UVB-treated samples were still higher than in controls (water-only treatment). These results suggest that preharvest UVB and calcium treatments boost the expression of genes for GL biosynthesis, but do not have much impact on the expression of those genes during postharvest storage. 

We also evaluated the expression of *MY* involved in GL breakdown during storage. On Day 0, there were no significant differences among controls (water only) and all other treatments in expression level of *MY* (Figure 4C). After cold storage, the expression levels of *MY* in controls on Day 7 and 14 were increased by about 24- and 28-fold, respectively, as compared to the expression levels observed on harvest day, indicating that GL degradation occurred dramatically during storage in these samples. However, *MY* levels in the samples treated with 10 mM calcium alone, UVB 0.27 Wh/m^2^ alone, and calcium plus UVB were much lower than those in controls, even though *MY* expression in all treatments was higher on Day 7 and 14 than on Day 0. For example, on Day 7 and 14, *MY* levels in UVB treated samples were 3.1- and 2.2-fold less, respectively, than in controls. No significant differences were observed between calcium and UVB treatments. Nonetheless, the samples treated with the combination of UVB and calcium displayed significantly lower *MY* levels on Day 7 and 14. Hence, *MY* expression levels correlated to the GL levels during storage (Table 1 and Figure 4). These results suggest that the reduction in GLs in broccoli microgreens during postharvest storage mainly resulted from *MY* catalyzed GL breakdown. UVB and calcium treatments might delay GL breakdown by reducing *MY* expression during postharvest storage. 

## 4. Conclusions

GLs are unique antioxidants produced in Brassica vegetables such as broccoli and show great health benefits for human health. These compounds are also regarded as signal molecules for plants to respond to oxidative stress caused by biotic and abiotic stresses. Our study indicates that preharvest UVB treatments plus 10 mM CaCl_2_ spray significantly elevated the concentrations of GLE, GLR, and total aliphatic GLs by around 70% in broccoli microgreens as compared to untreated controls. The nutritional qualities of the two groups of UVB-treated microgreens were stable during 21-day storage, with only small changes in their GL contents. The UVB 0.27 Wh/m^2^ with 10 mM CaCl_2_ spray-treated broccoli microgreens maintained their overall quality throughout shelf life, and showed the lowest TEL and off-odor values during the 14- and 21-day evaluations. Glucosinolate levels were found to have a significant positive correlation with overall quality, and negative correlation with off-odor in broccoli microgreens. Furthermore, UVB 0.27 Wh/m^2^ and/or calcium treatment of microgreens significantly reduced the expression of *MY*, a key gene responsible for GL breakdown during postharvest storage, suggesting that *MY* level could be used as a molecular marker to predict broccoli microgreen postharvest quality and shelf life. This study also provides an efficient and simple method to enhance the health-beneficial compounds in broccoli microgreens, which can be utilized by industry.

## Figures and Tables

**Table 1:** Glucoraphanin, glucoerucin and total glucosinolate contents of preharvest UVB-treated broccoli microgreens during storage.

	Glucoerucin (µmol/g)	Glucoraphanin (µmol/g)	Total (µmol/g)
Day 0 Control	28.89 ± 0.57 ^d^	1.51 ± 0.03 ^cd^	30.10 ± 0.58 ^d^
Day 0 10 mM CaCl_2_	39.07 ± 0.53 ^f^	1.68 ± 0.02 ^d^	40.75 ± 0.54 ^f^
Day 0 UV0.09	32.76 ± 0.59 ^e^	1.89 ± 0.03 ^e^	34.65 ± 0.58 ^e^
Day 0 UV0.27	33.76 ± 0.37 ^e^	1.93 ± 0.06 ^e^	35.69 ± 0.32 ^e^
Day 0 10 mM CaCl_2_ + UV0.09	46.60 ± 0.29 ^g^	2.00 ± 0.04 ^e^	48.59 ± 0.31 ^g^
Day 0 10 mM CaCl_2_ + UV0.27	48.86 ± 2.24 ^g^	2.01 ± 0.11 ^e^	50.87 ± 2.34 ^g^
Day 3 Control	23.21 ± 0.51 ^c^	1.32 ± 0.03 ^c^	24.53 ± 0.53 ^c^
Day 3 10 mM CaCl_2_	36.08 ± 1.34 ^ef^	1.64 ± 0.02 ^d^	37.72 ± 1.36 ^ef^
Day 3 UV0.09	31.79 ± 0.17 ^e^	1.87 ± 0.02 ^e^	33.67 ± 0.16 ^e^
Day 3 UV0.27	32.80 ± 0.98 ^e^	1.92 ± 0.02 ^e^	34.72 ± 1.00 ^e^
Day 3 10 mM CaCl_2_ + UV0.09	45.64 ± 0.59 ^g^	1.97 ± 0.05 ^e^	47.60 ± 0.56 ^g^
Day 3 10 mM CaCl_2_ + UV0.27	47.15 ± 1.88 ^g^	1.99 ± 0.05 ^e^	49.14 ± 1.84 ^g^
Day 7 Control	18.71 ± 0.33 ^b^	1.01 ± 0.02 ^b^	19.72 ± 0.34 ^b^
Day 7 10 mM CaCl_2_	34.06 ± 0.64 ^e^	1.54 ± 0.01 ^cd^	35.61 ± 0.66 ^e^
Day 7 UV0.09	29.42 ± 1.82 ^d^	1.78 ± 0.06 ^de^	31.20 ± 1.77 ^d^
Day 7 UV0.27	30.17 ± 0.18 ^d^	1.85 ± 0.07 ^e^	32.02 ± 0.25 ^d^
Day 7 10 mM CaCl_2_ + UV0.09	45.01 ± 0.83 ^g^	1.83 ± 0.07 ^de^	46.84 ± 0.77 ^g^
Day 7 10 mM CaCl_2_ + UV0.27	45.46 ± 1.58 ^g^	1.86 ± 0.03 ^e^	47.32 ± 1.56 ^g^
Day 14 Control	13.98 ± 0.22 ^a^	0.85 ± 0.09 ^a^	14.83 ± 0.30 ^a^
Day 14 10 mM CaCl_2_	27.52 ± 0.59 ^d^	1.37 ± 0.07 ^c^	28.90 ± 0.52 ^d^
Day 14 UV0.09	28.41 ± 1.75 ^d^	1.59 ± 0.08 ^cd^	30.00 ± 1.77 ^d^
Day 14 UV0.27	28.38 ± 0.33 ^d^	1.64 ± 0.05 ^d^	30.11 ± 0.38 ^d^
Day 14 10 mM CaCl_2_ + UV0.09	38.72 ± 0.81 ^f^	1.83 ± 0.07 ^de^	40.55 ± 0.79 ^f^
Day 14 10 mM CaCl_2_ + UV0.27	39.17 ± 0.47 ^f^	1.86 ± 0.03 ^e^	41.03 ± 0.45 ^f^
Day 21 Control	12.85 ± 0.41 ^a^	0.71 ± 0.05 ^a^	13.56 ± 0.45 ^a^
Day 21 10 mM CaCl_2_	23.25 ± 0.70 ^c^	1.14 ± 0.07 ^b^	24.39 ± 0.76 ^c^
Day 21 UV0.09	21.62 ± 0.38 ^c^	1.32 ± 0.07 ^c^	22.94 ± 0.44 ^c^
Day 21 UV0.27	22.62 ± 1.22 ^c^	1.43 ± 0.05 ^c^	24.05 ± 1.24 ^c^
Day 21 10 mM CaCl_2_ + UV0.09	34.44 ± 0.47 ^e^	1.41 ± 0.06 ^c^	35.85 ± 0.53 ^e^
Day 21 10 mM CaCl_2_ + UV0.27	36.15 ± 0.47 ^ef^	1.44 ± 0.03 ^c^	37.59 ± 0.43 ^ef^

All the data are expressed as Mean ± SD of three replicates followed by a letter. The same letter means no statistical difference whereas different letters indicate a significant statistical difference (*p* > 0.05).

## Data Availability

The authors declare that all data generated or analyzed during this study are included in this published article.
